# Functional and Structural Network Disorganizations in Typical Epilepsy With Centro-Temporal Spikes and Impact on Cognitive Neurodevelopment

**DOI:** 10.3389/fneur.2019.00809

**Published:** 2019-08-29

**Authors:** Emilie Bourel-Ponchel, Mahdi Mahmoudzadeh, Azeez Adebimpe, Fabrice Wallois

**Affiliations:** ^1^INSERM UMR 1105, Research Group on Multimodal Analysis of Brain Function, University of Picardie Jules Verne, Amiens, France; ^2^INSERM UMR 1105, EFSN Pediatric, Amiens University Hospital, Amiens, France

**Keywords:** benign epilepsy with centro temporal spike, neurocognitive impairment, interictal epileptic spike, high density EEG, time frequency analysis, connectivity

## Abstract

Epilepsy with Centrotemporal Spikes (ECTS) is the most common form of self-limited focal epilepsy. The pathophysiological mechanisms by which ECTS induces neuropsychological impairment in 15–30% of affected children remain unclear. The objective of this study is to review the current state of knowledge concerning the brain structural and functional changes that may be involved in cognitive dysfunctions in ECTS. Structural brain imaging suggests the presence of subtle neurodevelopmental changes over the epileptogenic zone and over distant regions in ECTS. This structural remodeling likely occurs prior to the diagnosis and evolves over time, especially in patients with cognitive impairment, suggesting that the epileptogenic processes might interfere with the dynamics of the brain development and/or the normal maturation processes. Functional brain imaging demonstrates profound disorganization accentuated by interictal epileptic spikes (IES) in the epileptogenic zone and in remote networks in ECTS. Over the epileptogenic zone, the literature demonstrates changes in term of neuronal activity and synchronization, which are effective several hundred milliseconds before the IES. In the same time window, functional changes are also observed in bilateral distant networks, notably in the frontal and temporal lobes. Effective connectivity demonstrates that the epileptogenic zone constitutes the key area at the origin of IES propagation toward distant cortical regions, including frontal areas. Altogether, structural and functional network disorganizations, in terms of: (i) power spectral values, (ii) functional and effective connectivity, are likely to participate in the cognitive impairment commonly reported in children with ECTS. These results suggest a central and causal role of network disorganizations related to IES in the neuropsychological impairment described in ECTS children.

## Highlights

- The epileptogenic zone and distant areas are structurally/functionally disorganized
- The functional disorganization is accentuated in the presence of IES on scalp EEG
- Distant desynchronization begins before IES
- A causal role of IES in ECTS neuropsychological impairment is highly suggested.

## Introduction

According to the international classification of epilepsy (1), Epilepsy with CentroTemporal Spikes (ECTS) is the most common form of self-limited, drug-responsive, focal epilepsy (2–4). ECTS accounts for 13 to 23% of all cases of new-onset epilepsy in children (5–8) with an incidence of about 10–20/100 000 children under the age of 13 years (3, 8). The peak age of onset of ECTS is 7 to 8 years and ECTS is slightly more common in boys, with a sex ratio of 60:40 (9).

### Clinical and Electroencephalographic Features of Typical ECTS

Diagnostic criteria are characterized by onset in childhood, absence of brain lesion on MRI neuroimaging, normal initial psychomotor development, normal pregnancy and delivery, sensorimotor seizures and focal centrotemporal or rolandic spikes and waves activated with sleep on inter-ictal EEG recording (2).

Neurological evaluation and cognitive development are normal, but cognitive impairments such as attention deficiency, visuomotor coordination impairment and specific learning disabilities have been described (see below).

Onset of seizures is usually between the ages of 4 and 10 years, and always before the age of 13 years (10). Seizures resolve by the age of 13 years. More than 90% of children experience active epilepsy for about 3 years (11). Seizures are uncommon, as more than 90% of children experience fewer than 5 seizures (12, 13) and they are usually closely related to sleep (55%) or appear during wakefulness or drowsiness (9). Only 30% of children experience seizures during wakefulness (2, 9). Eighty percent of seizures are focal with inconstant secondary generalization (13). Focal seizures involve sensorimotor areas. Motor signs affect the face (37%) and oropharyngeal muscles (53%), including guttural sounds, mouth movements, dysarthria and clonic jerks (40%) and may be preceded by sensory sensations such as hemifacial, perioral or intraoral paraesthesia, and jaw and tongue stiffness (13). The upper limb (20%) and rarely the lower limb (8%) are less frequently affected (13). Generalized seizures have been reported in 24–80% of patients (9, 13), but probably correspond to secondary generalization of focal seizures. Seizures usually last only a few minutes, and always, less than half an hour (9).

In typical ECTS, standard EEG shows centrotemporal spikes on normal background activity (8). Interictal Epileptic Spikes (IES) may be either isolated or occur in clusters and exhibit a high voltage biphasic wave with centrotemporal negativity followed by a positive frontal dipole (14–16). IES can be unilateral (60%) or bilateral (40%), synchronous or asynchronous between the two hemispheres (15, 17) and lateralization may change between different EEGs in the same patient. IES are frequently activated during non-REM sleep (15, 18–20) and diffuse in the ipsilateral and contralateral hemispheres (21). About 30% of patients only present spikes during sleep (18) EEG abnormalities resolve at puberty, but frequently persist after the period of seizures (11).

### Typical ECTS in the Spectrum of Rolandic Epilepsy

Typical ECTS belongs to the spectrum of rolandic epilepsy, which encompasses typical ECTS, Atypical Benign Partial Epilepsy (ABPE), Landau-Kleffner syndrome (LKS) and epileptic encephalopathy with Continuous Spikes and Waves during slow-wave Sleep (CSWS) (22).

ABPE is defined by atypical seizures (long partial or generalized motor, atonic seizures or negative myoclonus, atypical absences, perioral myoclonia) (23, 24) associated with peri-ictal symptoms, such as transient oro-motor dysfunction. Atypical patterns are observed on EEG, characterized by an intermittent slow-wave focus, multiple asynchrony spike-wave foci, long spike-wave clusters, and “absence-like” spike-wave discharges (25, 26). LKS and CSWS share several common features with typical ECTS: onset of epilepsy during childhood, remission before the end of adolescence, rare seizures, and normal brain MRI. EEG abnormalities are associated with deterioration of cognitive functions that were previously normally acquired. LKS is defined by acquired epileptic aphasia with verbal auditory agnosia and behavioral disorders associated with a marked increase of IES during sleep, predominantly in the temporal areas. CSWS is characterized by continuous spike-and-wave discharges during slow-wave sleep, usually combined with global intellectual deterioration and epileptic seizures (27).

Typical ECTS and rolandic epilepsy spectrum are believed to represent a continuum of different clinical phenotypes of self-limited focal epilepsy (28) but some authors consider them to be fundamentally distinct conditions (9, 15, 29–31).

### Genetic Origin of Typical ECTS

The genetic origin of ECTS was first suggested on the basis of clinical observations (32) comparing the incidence of ECTS in twins and families with sporadic ECTS (33–37). However, the direct causal role of monogenic mutations in typical ECTS has not been clearly demonstrated. In contrast with LKS and CSWS (38–42), monogenic mutations including GRIN2A (38, 39, 42), KCNQ2, KCNQ3 or duplications in PRRT2 gene (35, 43–48) have been detected in only a small proportion of children with typical ECTS. ECTS has therefore been attributed to a complex interplay between brain development, maturation processes and gene susceptibility with no evidence, at the present time, that genetic factors are paramount (49, 50).

### Neuropsychological Impairment in Typical ECTS

Normal cognition was previously considered to be a prerequisite for the diagnosis of ECTS, which is why this form of epilepsy was initially called Benign Childhood Epilepsy with Centro-Temporal Spikes (BCECTS) (ILAE, 1985; ILAE, 1989). However, ~15–30% of affected children show some degree of neuropsychological impairment, including disorders of language processing (51–55), attention (56–58), and executive functions (59), verbal function (52, 60–64), short-term (52, 54), and working memories (65), psychiatric status and general cognitive functioning (66, 67). These neuropsychological impairments impact academic achievement as a result of specific learning disabilities (62, 68, 69).

The pathophysiological mechanisms by which ECTS induces neuropsychological impairment remain unclear. The neuropsychological impairments are not related to seizure characteristics (26) and are observed even in children with well-controlled seizures (70–72). The impact of antiepileptic drugs has not been clearly determined (73). The role of IES has been suggested but remains debated. The rate of occurrence of IES in ECTS does not appear to be sufficient to predict the individual cognitive outcome (26, 58, 62, 68, 73). The morphology of interictal abnormalities appears to be predictive of neurocognitive and clinical outcomes, as combinations of various interictal EEG parameters (slow wave focus, multiple asynchronous spike and wave foci, long rhythmic spikes and clusters, generalized spike and wave discharges, abundance of interictal abnormalities during sleep and wakefulness, persisting for several months) precede clinical worsening, but these parameters are considered to be diagnostic criteria for ABPE (26). The localization of interictal discharges might be partly related to the type of cognitive deficit (74). Nevertheless, the correlation between IES localization on the EEG and the neuropsychological profile of children with ECTS has yet to be clearly defined (75, 76).

Despite the large number of studies conducted since the first description of ECTS by Nayrac and Beaussart (77), the pathophysiological mechanisms that participate in ECTS and relative neurocognitive impairment have yet to be defined.

The objective of this study is to review the functional and structural disorganizations in the epileptogenic zone, but also in remote areas, that may be involved in cognitive dysfunctions in patients with typical ECTS. Before addressing these disorganizations, we will review the current state of knowledge concerning the epileptogenic zone responsible for structural and functional interactions with distant regions.

## The Epileptogenic Zone and the Spike Onset Zone in ECTS

**Figure 1:**
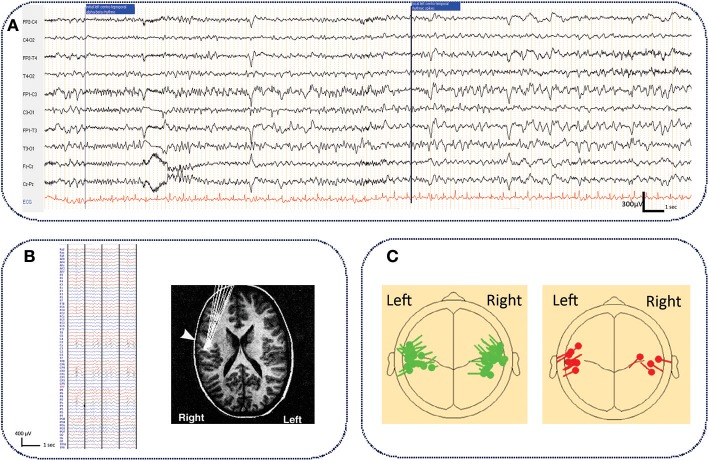
Typical ictal aspect on standard EEG in ECTS and electrical source imaging of IES in typical and atypical ECTS. **(A)** Typical aspect of ECTS seizure characterized by initial focal low amplitude alpha-beta activity (9–14 Hz), gradually increasing in amplitude and decreasing in frequency to the alpha band (6–8 Hz), followed by rapid rhythmic spikes. Longitudinal bipolar montage, electrodes positioned according to the international 10/20 system. Band pass (0.53–70 Hz), notch filter (50 Hz) (personal data). **(B)** Typical right centro-temporal IES on normal background activity recorded with HD EEG [averaging referential montage, band pass (0.53–70Hz)] and electrical source imaging (HD EEG) of IES with tangential dipoles located along the central sulcus with the negative pole situated in the posterior mid-temporal or central regions [*adapted with authorization from Bourel-Ponchelet al*. (83)]. **(C)** Comparison of typical (in green) and atypical generator (in red) of IES in ECTS and Atypical Benign Partial Epilepsy, respectively. Compared to ECTS, in Atypical Benign Partial Epilepsy, IES dipoles have a preferential posterior orientation *[reproduced with authorization from Kim et al*. *(**84**). Averaged EEG spike dipole analysis may predict atypical outcome in Benign Childhood Epilepsy with Centrotemporal Spikes (BCECTS). Brain Dev. 38, 903–908]*.

*The epileptogenic zone* is defined by the site of onset of epileptic seizures and their primary organization (78). Due to the rarity of seizures and the absence of intracranial recordings, the epileptogenic zone is difficult to localize in ECTS. In rare ictal scalp EEG recordings (25, 79–82), the onset of focal seizures was predominantly observed in the centro-temporal region (82), and occasionally in the parietal region (25) (Figure 1A). Despite small differences according to the epileptogenic zone, all EEG recordings show a similar pattern characterized by low amplitude focal alpha-beta activity (9–14 Hz), gradually increasing in amplitude and decreasing in frequency to the alpha band (6–8 Hz), followed by rapid rhythmic spikes (79). This ictal pattern mostly occurs after an increment of interictal spikes (79) together with a reversal of the IES dipole (81). An ictal electrical source imaging study also demonstrated an operculo-insular origin of the initial rhythmic ictal activity (79).

*The spike onset zone* is defined by the site of generation of the IES. The epileptogenic zone is usually included in the spike onset zone (78). In ECTS, as in all forms of focal epilepsy, the epileptogenic zone could be approximated by the spike onset zone.

Studies using voltage gradient measures applied to IES in ECTS have defined the spike onset zone in the mid-centro-temporal region (85). Using Electrical Source Imaging, the source of the IES has been modeled by a single tangential dipole located along the central sulcus. This dipole is oriented tangentially to the surface of the scalp (86–88). The negative pole is situated in the posterior mid-temporal or central regions and a positive pole is centered more anteriorly, involving frontal areas (83, 88–90) (Figure 1B). Although the posterior side of the central sulcus was initially believed to generate the IES (90, 91), recent studies have proposed that the IES originate from the rolandic fissure of the somatosensory cortex (area 3b) (88–90, 92). More precisely, superior finger/hand areas have been proposed as a possible origin of IES, which may then propagate and extend along the precentral sulcus to the mouth/tongue area (93). Atypical generator characteristics have been proposed as criteria to help clinicians to rapidly discriminate typical ECTS from ABPE in individual patients (Figure 1C) (84, 94, 95). In these atypical generators, the dipoles have a preferential posterior orientation (84). Source locations are not tightly clustered around the central sulcus (95) and could be located around the rolandic sylvian regions, involving the motor cortex (91, 96) or even more lateral and inferior areas than those reported in typical ECTS (86).

## Structural Disorganization in ECTS

As ECTS is defined as an idiopathic or self-limited epilepsy syndrome, no MRI abnormalities are expected. However, subtle structural abnormalities have been described in the epileptogenic zone and in distant brain structures, suggesting that the epileptogenic processes interfere with normal brain development and maturation.

During normal neurodevelopment and under physiological conditions, the gray matter volume increases until preadolescence and decreases thereafter, whereas the volume of white matter increases until adulthood (97–99). Synaptic exuberance and pruning are thought to contribute to these morphological changes (100, 101). Post-natal brain development is associated with the generation of excess neuronal synapses. In immature animals, the number of axonal branches, development of the dendritic spine and synaptic connections exceed those observed in mature animals. Connections may exist between parts of the brain that are not interconnected in mature animals. The generation of excess neuronal synapses is followed by decline until adulthood throughout the cerebral cortex (102–106). This selective elimination of synapses, called pruning, depends on synaptic activity and promote the maintenance of more active synapses, while removing less active synapses. Pruning is thought to be necessary to refine the emerging brain circuitry (107–110).

ECTS could impact this ongoing neurodevelopment in various ways. In newly diagnosed ECTS, neuroimaging studies have revealed differences (increases or decreases) in the thickness of the cortex (Table 1). These changes are not specifically observed in the central epileptogenic zone, as they have been observed in distant areas, notably in the frontal and temporal regions (112–115, 118) and in subcortical structures, especially the putamen (111, 114, 117). ECTS children also demonstrate white matter abnormalities [reduced fractional anisotropy (FA)] over the central epileptogenic zone (120) and over distant regions (the splenium of the ipsilateral corpus callosum) (113, 121). These differences in brain volume and brain structural connectivity suggest the presence of subtle neurodevelopmental changes between typical development in normal children and those with ECTS. This structural remodeling likely occurs prior to the diagnosis of ECTS following onset of the first seizure (61, 115, 118, 119).

**Table 1:** Structural brain disorganization in ECTS.

**Study**	**BECTS (*n* =)**	**Control (*n* =)**	**Sub-cortical gray matter**				**Cortical gray matter**					**Growth disturbance**	**Cognitive correlation**
			**Putamen**	**Caudate nucleus**	**Amygadala**	**Thalamus**	**Frontal**	**Temporal**	**Parietal**	**Occipital**	**Cingular gyrus**		
Lin et al. (111)	13	54	↑^*^			=^*^							Executive performance
Hermann et al. (112)	38	34					=^*^	=^*^	=^*^	= ^*^		No	
Kim et al. (113)	20	20	↑^*^		↑^*^		↑ R sup $ ↑ L orbito-front ^*^ ↑ L pars orbitalis ^*^ ↑ L precentral ^*^	↑ R sup $ ↑ R middle $	↑ R pre-cuneus ^*^		↑ L ant ^*^ ↑ L post ^*^		ADHD
Luo et al. (114)	21	20	↑^*^				↑ R SMA ^*^ ↑ R Ant insula ^*^ ↑ R operculum ^*^ ↑ B Paracentral lobule ^*^	↑ R Inf ^*^					
Pardoe et al. (115)	35	35					↑ B sup ^*^$ ↑ B insula ^*^$ ↑ B inf ^*^$		↑B supra-marginal $			Yes (1)	
Overvliet et al. (61)	24	24						↓ L sup $	↓ L supra- marginal $ ↓ L postcentral $			Yes (2)	No
Saute et al. (116)	18 (ADHD+) 36 (ADHD -)	46					ADHD +: ↓ L insula $ ↓ R sup $ ↓ L Paracentral $ ↓ R pars opercularis $	↓ L middle left $ (ADHD-)	↓ L inf $ ↓ L sup $				ADHD
Shakeri et al. (117)	41	38		↑ R (3) ^*^									
Garcia-Ramos et al. (118)	24	41	↑^*^				↓ B rostral middle ^*^ ↓ R inf ^*^			↓ L lat ^*^		Yes (4)	
Kanemura et al. (119)	2 (CI+) 5 (CI-)	11										Yes (5)	Yes

Neuroimaging studies demonstrated structural disorganization in cortical and sub-cortical structures in newly diagnosed ECTS, which evolved with the brain development. Functional correlations between structural remodeling and cognitive deficits have been inconsistently addressed. R, right; L, left; B, bilateral; sup, superior; ant, anterior; post, posterior; front, frontal; SMA, supplemental motor area; inf, inferior; lat, lateral; ADHD, Attentional Deficit with Hyperactivity Disorder; CI, cognitive impairment; ↑, increase; ↓, decrease;

* *Volume gray matter, $ cortical thickness mapping. (1) Normalization with the age, (2) left-lateralized frontal, centro-parietal and temporal regions with pathological thinning with the age, (3): in case of bilateral IES, (4) Pathological thickening and thinning with the age (5) deficit of frontal and prefrontal lobe volume growth in case of cognitive impairment*.

Some studies have reported that this structural remodeling evolves over time (61, 115, 118, 119), as, during the active phase of ECTS (persistence of seizures and IES on interictal EEG), differences in cortical and subcortical thickness have been progressively observed compared to healthy age-matched controls, especially in patients with cognitive impairment (111, 115, 118, 120, 122) (Table 1). Following ECTS remission (115, 119), changes in gray matter growth, notably in the frontal lobe, tend to resolve, with more rapid recovery in ECTS patients with a shorter active seizure period (123) (Table 1). The epileptogenic processes related to synaptic hyperactivity and/or alterations of physiological pruning might affect the dynamics of the brain development and/or might interfere with the normal maturation processes (61, 109, 115, 118–120, 122, 124, 125).

Functional correlations between structural remodeling and cognitive deficits have been recently addressed (61, 113, 116, 119). A correlation between ADHD in ECTS children and structural impairment in the cortico-striatal circuitry has been inconsistently reported (113, 116). Similarly, a decrease in frontal and pre-frontal gray matter volume was more pronounced in ECTS with cognitive impairment and behavioral issues (119) (Table 1).

## Functional Disorganization: From the Epileptogenic Zone to Distant Networks

### Functional Disorganization in the Epileptogenic Zone

**Figure 2:**
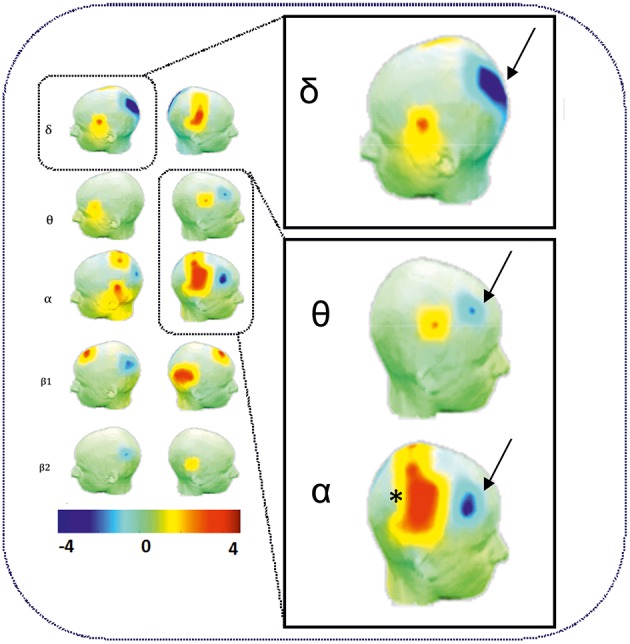
Power spectrum analysis of functional disorganization in the epileptogenic zone in remote regions. **(Right Top)** Increase in δ, θ, and α power spectra (arrows) in the epileptogenic zone over the centro-temporal areas in the presence of IES. **(Right Bottom)** Relative decrease in power value (arrows) in θ and α frequency bands in frontal areas and in δ bands in occipital areas. Statistical difference (*t*-value) maps of degree differences between HD EEG segments with IES and HD EEG segments without IES. The color bar indicates the t values projected onto a standardized head shape. The significant increase (indicated by red) and decrease (indicated by blue) in degree have been represented by positive and negative t values resulted from statistical comparisons between IES condition and no IES condition *[adapted from Adebimpe et al*. *(**127**), reproduced under the Creative Commons CC-BY license]* (126).

**Figure 3:**
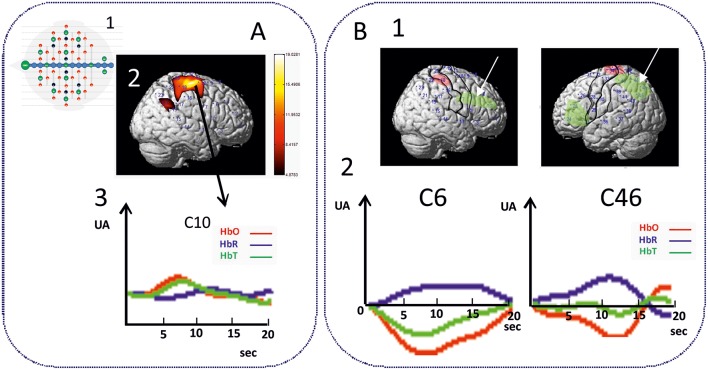
Hemodynamic response of functional disorganization in the epileptogenic zone **(A)** in remote regions **(B)**. A1 High density EEG-fNIRS drawing of the bimodal EEG-fNIRS cap with optodes (detectors, green; emitters, red) and electrodes (black) positions. Twenty three channels per hemispheres have been considered corresponding to distances between emitting and detecting fibers (1.5 to 4 cm). A2 Hemodynamic responses related to right centro-temporal IES in a patient with typical ECTS. Statistical map of the hemodynamic response using a typical Hemodynamic Response Function (*p* < 0.05) and (3) Traces of the hemodynamic response after averaging (0-20 seconds) of the related channel 10 (C10). IES in ECTS are associated with a typical positive hemodynamic response, as demonstrated by EEG-fNIRS in the centro-temporal area [*adapted from Bourel-Ponchel et al*. [*130*]]. B1 Positive and negative neurovascular coupling areas (in red and green, respectively) calculated from a typical Hemodynamic Response Function (HRF) (*p* < 0.05). B2 Negative neurovascular coupling obtained with averaging method (range considered 0–20 s) in right frontal and left parieto-occipital areas (arrows). High density optical imaging found increase in HbR and decrease in HbO (corresponding to a negative BOLD) in the same regions (bi-frontal areas and left occipital areas) (136). x Axis: time range between 0 and 20 s (interval between vertical bars: 5 sec). y Axis: arbitrary unit (UA): relative concentrations variations related to IES. Red curve: HbO, Blue curve: HbR, Green curve: HbT.

As expected, an increase in θ, α, and ß power spectra and cortical source densities (sensor space) (126) has been observed in the epileptogenic zone over the centro-temporal areas in the presence of IES (Figure 2). This increase in power spectrum over the centro-temporal areas was accompanied by higher phase synchronization when comparing EEG segments with and without IES in ECTS children (126, 128). In parallel, High frequency Oscillations (HFOs) (50–250 Hz) superimposed on top of IES were described in typical and atypical ECTS (129–131). The hypersynchronization due to IES is associated with increased local hemodynamic changes, as demonstrated by EEG-functional MRI (132–135) and EEG-functional Near InfraRed Spectroscopy (EEG-fNIRS) (136) (Figure 3A).

**Figure 4:**
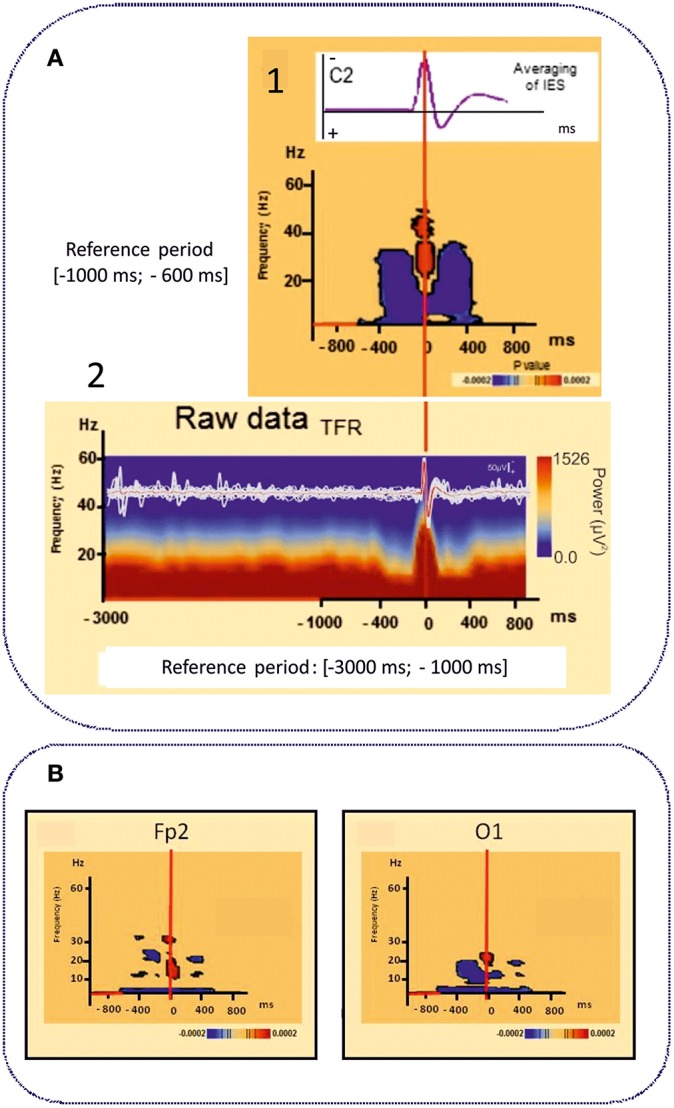
Time frequency analyses of functional disorganization in the epileptogenic zone **(A)** in in remote regions **(B)**. A1 Averaging of the selected right centro-temporal IES in typical ECTS at electrode C2 [HD EEG, 64 electrodes positioned according to the 10/10 international system, band pass (0.53 −15 Hz), notch filter (50 Hz)]. Bellow, Time frequency statistical analysis at electrode C2 for the same patient [reference period (−1000; −600 ms), (*p* < 0.0002)]. A2 Raw data of the time frequency analysis [Reference period: (−3000 ms −1000 ms)] for the same patient. T0 was defined as the first negative deflexion of the spike. Time-frequency analysis revealed complex sequences of desynchronization (in blue) -synchronization (in red) -desynchronization (in blue) surrounding the IES in ECTS for frequencies range from 4 to 50 Hz independently of the baseline considered [−1000; −600 ms] (1) or [−3000 ms; −1000 ms]. This desynchronization was localized near the epileptogenic zone *[adapted from Bourel-Ponchel et al*. *(**83**), reproduced under the Creative Commons CC-BY license]* (83). **(B)** Time frequency representation of desynchronizations (in blue) occurring, in frontal and occipital areas, distant to the epileptogenic zone located in central area, involving low frequency bands (bellow 10 Hz), in the same time window as local alternation of synchrony around the IES (Figure 2) [−400 ms; +400 ms]. Significant statistical (*p* < 0.0002) results of time frequency analysis at electrodes Fp2 and O1 for the same patient (reference period [−1000; −600 ms] *[adapted from Bourel-Ponchel et al*. *(**83**)], reproduced under the Creative Commons CC-BY license)* (83).

However, IES can no longer be simply considered to be due to hypersynchronization of a single homogeneous population of neurons. Using time-frequency analysis, complex sequences of desynchronization-synchronization-desynchronization have been demonstrated surrounding the IES in ECTS (83) (Figure 4A). These sequences began 400 ms before the IES, suggesting complex disorganization of the network in the epileptogenic zone starting before the onset of the neuronal network process of the IES as recorded by EEG. These phenomena are not specific to ECTS, as they have also been described in animal models of epilepsy (137) and in non-idiopathic frontal epilepsy in children (138) and could be explained by various patterns of neuronal activation and deactivation occurring 400 ms before hypersynchronization (139). Interestingly, these swings in synchronization are observed in parallel with swings in membrane properties observed on Fast Optical Signal (FOS) recordings in animals and children with frontal epilepsy, which might result in sequences of shrinking, swelling and shrinking of the neuronal population involved in the IES process. These non-synaptic events around the IES likely modify the extracellular space and consequently the bioavailability of neurotransmitters that propels neurons to hypersynchronization (137, 138).

Higher relative EEG power in the θ, α, and ß bands is still observed in the centro-temporal area regardless of the presence of IES in the EEG segments (126). Profound changes in terms of spectral power (126), phase synchronization (140), network degree and clustering coefficient (127, 141, 142) are observed simultaneously in the epileptogenic zone, independently of the presence of IES on EEG (Figure 2).

Altogether, these results indicate profound disorganization of the epileptogenic zone, not only in the time-window around the IES, but also during the period during which IES are not detected. IES are transient biomarkers of the local complex disorganization that is consubstantial to the epileptic network in ECTS. This disorganization is effective several hundred milliseconds before the IES (83) and consists of aberrant local synchronization/desynchronization and higher levels of neuronal activity (142–144) that do not always reach the threshold of an IES recorded on the scalp EEG.

### From the Epileptogenic Zone to Distant Networks

#### Involvement of Distant Areas

Functional MRI and HD-EEG studies in the interictal state, regardless of the presence of IES, have demonstrated profound changes of the spectral power in distant areas to the spike onset zone (Figures 2, 4B). Desynchronization in the α and ß bands observed in bilateral frontal and parieto-occipital areas, suggests disengagement of the frontal and parieto-occipital cortices (127, 140, 142). Using fNIRS and fMRI (132, 133, 136), the hemodynamic response to IES (decrease in HbO, increase in HbR) in distant areas, including the frontal and parieto-occipital lobes, confirmed the widespread effect of IES on remote networks (Figure 3B).

#### Functional Connectivity

Functional connectivity measurements have been used to study functional alteration of the brain networks during the interictal state.

In the human brain, inhibitory and excitatory circuits interact by integrating information at local and global levels. It has been shown that the normal brain has a small-world functional topology, which can efficiently combine functionally specialized (segregated) modules with intermodular links (integrating). This type of organization reflects an optimal balance between functional integration and segregation (145, 146). This small-world functional topology is disrupted in ECTS.

A higher connection density around the epileptogenic zone, including the motor areas, the central region and the ipsilateral temporal region, has been described in all frequency bands (126). Functional connectivity using Lagged Phase Synchronization (LPS) analysis revealed higher θ and α and lower β LPS values (141). Functional connectivity changes around the IES were associated with lower segregation and higher integration, notably in the θ and α bands, compared to controls, suggesting that the ECTS brain network differs from the small-world features observed in healthy controls in a frequency-dependent manner. Loss of global processing and stronger integration have been observed in the epileptogenic zone, but also between remote brain regions, notably the frontal, parietal and temporal lobes (126, 127, 140–142, 147). These results indicate that the ECTS brain networks are less well-organized regardless of the presence or absence of IES and regardless of the frequency band, corresponding to alteration of global small-world properties toward a more random network (126).

#### Dynamics and Directionality of Interictal Connectivity

The direction of information flow between the various brain regions has also been studied in ECTS children using effective connectivity analysis. Tools such as Dynamic Causal Modeling (DCM) can be used to measure the effective connectivity between selected brain regions (central epileptic region, temporo-parietal junction, temporal pole and precuneus) (148). This study showed that central rolandic regions constitute the key zone of origin of IES propagation in ECTS. DCM analysis of the direction of flow of information between brain regions from a causal perspective showed that the central epileptogenic zone exerts a greater causal influence on ipsilateral and contralateral distant cortical areas including the prefrontal cortex, temporo-parietal junction and temporal pole (148, 149). In other words, changes in distant cortical regions, notably at the frontal and temporo-parietal junction and in the temporal pole, are driven by the epileptogenic zone (148, 150). Moreover, in an fMRI study, Wu et al. showed, in addition to the driving effect from the epileptogenic zone to cortical areas, a causal link from the epileptogenic zone to subcortical structures (bilateral putamen, caudate nuclei) and cerebellum (149).

Functional changes in terms of power spectral values, functional and effective connectivity and small-world properties can participate in the cognitive impairments commonly reported in children with ECTS. The effect of pathological epileptic HFO on cognitive function needs also to be considered.

The epileptogenic zone induces functional changes in the frontal lobes, which play a major role in the processing and execution of higher cognitive skills and behavior (151). The centro-frontal pathway is altered in ECTS patients with an abnormal information flow arising from central areas during IES. Frontal deactivation and disturbances of connectivity are correlated with cognitive performances (140) and might explain learning, memory and attention difficulties in ECTS patients (73, 152–155). The causal link from the central to the frontal areas constitutes a source of abnormal information flow onto the frontal structures, suggesting that IES may play a role in alteration of the attention network (148). In addition to functional changes involving the centro-frontal network, decreased functional connectivity in the dorsal attention network (intraparietal sulcus and frontal eye fields of each hemisphere) associated with increased functional connectivity in the ventral attention network (temporo-parietal junction and ventral frontal cortex) might also be responsible for the attention deficit observed in ECTS (155). Functional connectivity disorders with the right inferior temporal cortex and bilateral primary auditory cortex may affect auditory processing in both hemispheres, resulting in language processing and speech processing deficits in ECTS patients (54, 141, 147, 156–158). The temporo-parieto-occipital areas are believed to be involved in auditory, visual, somatosensory and memory processes that can be impaired in ECTS children (142, 143). Finally, the reduced degree in occipital areas may explain the poor visuospatial memory observed in ECTS children (126, 159).

## Cognition and Brain Maturation in Children With ECTS

In summary, the available literature demonstrates the existence of:

1. Complex functional disorganizations in the epileptogenic zone that are accentuated in the presence of IES on scalp EEG
2. Complex functional changes in terms of activity and synchronization in bilateral distant networks that are accentuated in the presence of IES on scalp EEG
3. Complex functional changes in distant areas beginning around 400 ms before the IES in the same time window as complex modifications of synchronization around IES (-400 ms; +400 ms) in the epileptogenic area
4. Complex functional connectivity changes including small-world network impairment
5. Impaired structural brain maturation in children with ECTS with no specificity for the epileptogenic area.

Altogether, these results suggest a central and causal role of the functional and structural disorganizations of the neuronal network that promote the IES in the neuropsychological impairment described in children with ECTS. Two non-exclusive hypotheses can be proposed to explain the pathophysiological mechanisms of the neuropsychological impairment. First, disorganizations related to IES might directly induce transient inhibition of the networks. Second, disorganizations related to IES might induce long-lasting effects on brain functioning and maturation.

### Disorganizations of the Neuronal Network Related to IES-Induced Transient Network Inhibitions

This hypothesis is supported by clinical observations of transient cognitive impairment (TCI). Transient cognitive impairment (TCI) (160, 161) is characterized by brief temporary deficits of attention, visuospatial memory and learning strategies related to IES on EEG. TCI is observed during the active phase of ECTS and resolves after resolution of the EEG abnormalities (62, 162, 163). TCI is considered to be the direct consequence of IES on neuronal processing (74, 132, 158, 164, 165). TCI is likely related to transient disruption of brain function occurring around the IES in the epileptogenic zone and in distant functional networks. Time-frequency analyses have demonstrated that interferences in the epileptogenic areas and distant areas start 400 ms before IES (83). Interestingly, TCI started 100–200 ms before the IES (166) and can last for up to 2 s following a focal IES (160, 167–169), suggesting a direct link between complex changes in terms of synchronization around IES and TCI.

### Disorganizations of the Neuronal Network Related to IES Induces Long-Lasting Effects on Brain Functioning and Maturation

In addition to the short-lasting effects, network disorganizations related to IES may have long-term effects on developing neural networks (170, 171).

This abnormal brain neurodevelopment could be related to reports that certain neuropsychological deficits persist despite return to a normal EEG and clinical remission (62, 162, 163), suggesting cumulative effects of IES on functional maturation of neuronal networks (149, 172–176). The early onset, at a critical period of maturation and the duration of active epilepsy should be considered when assessing the functional outcome and cognitive prognosis. Similarly, the potentiation of IES during NREM sleep needs to be considered. The degree of cognitive impairment is correlated to the amount of NREM sleep-related IES and associated with the involvement of a widespread cortico-subcortical network related to IES during (177). In addition, the rate of pathological epileptic HFO during NREM sleep seemed to be a marker of epilepsy severity. HFOs rate is higher in atypical BECTS and Electrical status epilepticus in sleep. Absence of HFO may predict a relative benign clinical entity, whereas in the presence of several ripples, the child is likely to have more seizures (129–131, 178).

Structural and functional disorganizations not related to IES but participating in neurocognitive impairment in ECTS should not be excluded.

## Conclusion

The complexity of the interactions between the epileptogenic network and remote functional networks has been demonstrated in ECTS. Structural brain imaging suggests the presence of subtle neurodevelopmental changes over the epileptogenic zone and over distant regions in ECTS. Functional brain imaging demonstrates profound disorganization accentuated by IES in the epileptogenic zone which are effective several hundred milliseconds before the IES. In the same time window, desynchronization and changes in neuronal activity are observed in bilateral distant networks. These functional changes are associated with the alteration of global small-world properties toward a more random network; the direction of information flow between the brain regions demonstrates that the epileptogenic zone constitutes the key area at the origin of IES propagation toward distant cortical regions.

Altogether, these results support the idea that disorganizations of the neuronal network related to IES may disrupt local and distant networks with a possible impact on functional and the maturational processes. If the impact of IES on cognitive impairments remains debated, these results suggest a central and causal role of network disorganizations related to IES in the neuropsychological impairment described in ECTS children. These results suggest that therapeutic suppression of IES should reduce the risk of neuropsychological impairment in children with ECTS. However, the currently available antiepileptic drugs are not suitable and, specific treatments for disorganizations of the neuronal network related to IES need to be developed.

## Author Contributions

EB-P, MM, AA, and FW conceived and designed the review, read and accepted the manuscript. EB-P, MM, FW wrote the paper.

### Conflict of Interest Statement

The authors declare that the research was conducted in the absence of any commercial or financial relationships that could be construed as a potential conflict of interest.

## Abbreviations

ABPE: Atypical Benign Partial Epilepsy
BCECTS: Benign Childhood Epilepsy with Centro-Temporal Spikes
CSWS: epileptic encephalopathy with Continuous Spikes and Waves during slow-wave Sleep
DCM: Dynamic Causal Modeling
ECTS: Epilepsy with Centrotemporal Spikes
FA: Fractional Anisotropy
HD EEG: High Density Electroencephalography
HFO: High frequency Oscillations
IES: Interictal Epileptic Spikes
LKS: Landau-Kleffner Syndrome
LPS: Lagged Phase Synchronization
MRI: Magnetic Resonance Imaging
fNIRS: functional Near InfraRed Spectroscopy
REM: Rapid Eyes Movements
TCI: Transient Cognitive Impairment.
